# Improvement of mask R-CNN and deep learning for defect detection and segmentation in electronic products

**DOI:** 10.1371/journal.pone.0329945

**Published:** 2025-09-08

**Authors:** Longjian Guo, Jianming Meng, Wei Hao, Deepak Kumar Jain

**Affiliations:** 1 Department of Electronic and Communication Engineering, Shandong College of Electronic Technology, Jinan, China; 2 Symbiosis Institute of Technology, Symbiosis International University, Pune, India; University of Baghdad, IRAQ

## Abstract

With the rapid development of industrial automation and intelligent manufacturing, defect detection of electronic products has become crucial in the production process. Traditional defect detection methods often face the problems of insufficient accuracy and inefficiency when dealing with complex backgrounds, tiny defects, and multiple defect types. To overcome these problems, this paper proposes Y-MaskNet, a multi-task joint learning framework based on YOLOv5 and Mask R-CNN, which aims to improve the accuracy and efficiency of defect detection and segmentation in electronic products. Y-MaskNet combines the high efficiency of YOLOv5 in target detection with the fine segmentation capability of Mask R-CNN and optimizes the overall performance of the model through a multi-task learning framework. Experimental results show that Y-MaskNet achieves a significant improvement in detection and segmentation tasks, with mAP@[0.5:0.95] reaching 0.72 (up from 0.62 for YOLOv5 and 0.65 for Mask R-CNN) on the PCB Defect Dataset, and IoU improving by 7% compared to existing methods. These improvements are particularly notable in small object detection and fine-grained defect segmentation, making Y-MaskNet an efficient and accurate solution for defect detection in electronic products, offering strong technical support for future industrial intelligent quality control.

## Introduction

With the rapid development of electronic product manufacturing, defect detection and quality control have become essential to ensure product performance and reliability. Particularly in high-precision electronic components such as circuit boards, chips, and integrated circuits, even minor defects can lead to performance instability or failure. Traditional defect detection methods [37,38], such as manual inspection and classical image processing techniques (e.g., edge detection, threshold segmentation), often struggle with complex backgrounds, small defects, and real-time data processing needs, resulting in high labor costs and low accuracy.

Recent advancements in deep learning have greatly improved defect detection, particularly in computer vision. Models like Mask R-CNN have shown excellent performance in instance segmentation by using Region Proposal Networks (RPNs) and Fully Convolutional Networks (FCNs) [36]. However, Mask R-CNN faces limitations, particularly in handling small object defects, background noise, and computational complexity. On the other hand, YOLOv5, a fast and efficient target detection model, excels in real-time performance but lacks fine-grained instance segmentation capabilities [39,40]. This paper proposes combining the strengths of YOLOv5’s fast detection and Mask R-CNN’s precise segmentation to create an efficient and accurate defect detection and segmentation system.

In view of the existing deep learning models in the field of defect detection of electronic products, this paper innovatively constructs a hybrid model architecture Y-MaskNet, which combines the efficient target detection ability of YOLOv5 and the fine instance segmentation ability of Mask R-CNN [41], and constructs a multi-task joint learning framework to solve multiple challenges in the field of defect detection of electronic products, including small object detection, background noise processing, model real-time and segmentation accuracy [42,43]. Specifically, YOLOv5 is used to quickly detect the defective region in electronic products, and Mask R-CNN is used to perform fine segmentation on the defective region, which improves the accuracy and detail precision of defect detection [44]. Through the joint optimization of loss functions of target detection task and segmentation task, Y-MaskNet can improve the accuracy and robustness of target detection while maintaining high speed [45].

The research in this paper fills the gap between the existing techniques for balancing high efficiency and high accuracy in defect detection of electronic products, proposes a new hybrid model framework, and experimentally demonstrates the superior performance of this model in defect detection and segmentation tasks of electronic products. The core innovation of this paper is to combine the advantages of YOLOv5 and Mask R-CNN, which improves the robustness and accuracy of the model under complex backgrounds and tiny defects through a multi-task learning framework, and provides a new technological solution for quality control and automated production of electronic products.

## Literature review

### Anomaly detection techniques

Recent advancements in anomaly detection have significantly improved defect detection in electronic products [9,10]. Traditional methods, such as statistical techniques, are limited in handling high-dimensional and nonlinear data [11]. In contrast, deep learning models, particularly Autoencoders and GANs [12,13,34], have shown great promise in identifying complex defects. Autoencoders detect anomalies by measuring reconstruction errors, while GANs generate realistic data while learning anomaly patterns [14,15]. These methods excel in detecting defects in complex backgrounds and small objects, making them more suitable for modern defect detection tasks.

However, one challenge faced by deep learning anomaly detection methods is the scarcity and imbalance of data. Especially in the case of small datasets of specific defects in some electronic products [16], how to effectively perform unsupervised learning and reduce the reliance on a large amount of labeled data is an important research topic in this field. In the future, how to further improve the generalization ability of the model in data-scarce scenarios and reduce the overfitting phenomenon is still a direction worth exploring.

### Multimodal learning with cross-domain applications

With the increasing complexity of defect detection requirements in electronic products, a single data source can no longer meet the requirement of accurately identifying defects. Multimodal learning [17], by fusing information from different data sources (e.g., images, acoustic data, sensor data, etc.), provides new ideas for improving the accuracy and robustness of defect detection. In practical applications, the combination of image and sensor data can describe the state of the product more comprehensively [18,19,35]. For example, by combining vision images and sensor data such as temperature and pressure, internal defects in circuit boards or other electronic components can be detected more accurately, especially in the identification of tiny cracks or subtle bubbles, where single modality is often not sufficient.

Cross-domain applications are also an important direction in multimodal learning. By borrowing advanced techniques from other domains (e.g., medical image processing, autonomous driving, etc.) [20], it can provide valuable insights for defect detection in electronic products. For example, in medical image analysis, important breakthroughs have been made in segmentation processing techniques for small objects and details, which can be migrated to defect detection in electronic products for fine defect recognition and segmentation [21]. Meanwhile, cross-domain transfer learning can also effectively alleviate the problem of insufficient defect data for electronic products, especially when the defect types are complex and the amount of data is limited [22], by migrating the labeled data from other industries, it can accelerate the model training and improve the detection accuracy.

Although multimodal learning and cross-domain applications offer significant advantages, there are still challenges in data fusion and feature alignment. How to maximize the synergistic effect between different data sources and how to deal with data inconsistency and noise between modalities remain core issues that need to be addressed [23]. In the future, researchers need to conduct in-depth explorations on data preprocessing, feature fusion techniques, and cross-domain migratory learning in order to better enhance the performance of models in complex detection tasks.

### Advances in deep learning for defect detection in electronic products

The application of deep learning in defect detection in electronic products, especially convolutional neural networks (CNNs), has made significant progress in recent years [24]. While traditional image processing techniques often require the manual design of features, deep learning models are able to automatically extract effective features from data [25], greatly simplifying the processing process.CNNs are particularly suitable for extracting spatial features from images, and are effective in identifying various types of defects, such as cracks, soldering problems, missing components, etc., in the defect detection of electronic products [26].

Currently, most of the deep learning-based defect detection methods use an end-to-end learning framework, which is capable of detecting and localizing defects at the same time [27,28]. Compared to traditional methods, deep learning not only improves the accuracy of detection, but also handles more complex defect patterns such as tiny cracks and surface defects. However, existing models still face the problems of insufficient accuracy and high data quality requirements in some cases, especially when there are many types of defects and data labeling is difficult [29]. To address these problems, researchers have proposed various approaches such as data augmentation, semi-supervised learning, and migration learning techniques to compensate for the lack of data and improve the generalization ability of the models.

Finally, deep learning offers a new way of thinking for defect detection in electronic products, and its advantages in accuracy and automation are beginning to be realized [30]. With the development of technology, how to enhance the robustness of the model and reduce the dependence on large labeled data [31], and how to improve the real-time and interpretability of the model in the production environment will be the research focus in the future.

## Materials and methods

### Model design and methods

The Y-MaskNet model integrates the strengths of YOLOv5 and Mask R-CNN, and employs a multi-task joint learning framework for the detection and segmentation of electronic products. The structure of the model employs multiple collaborative modules that interact with each other tightly in the whole process from input to output to complete efficient processing and accurate segmentation of electronic product images.

Firstly, the input electronics images pass through a shared feature extraction network. YOLOv5 acts as the backbone network, which employs an efficient Convolutional Neural Network (CNN) structure to extract deep features in the image and perform rapid target detection.YOLOv5 target detection head is used to locate the defective regions in the image and generate the bounding boxes and category prediction for each target in the image. The design of this part is focused on fast localization and efficient detection to meet the requirement of rapid localization and initial defect recognition in the large-scale production line.

After finishing the target detection, the target candidate regions output from YOLOv5 pass to Mask R-CNN for fine instance segmentation.The Mask R-CNN further refine the target candidate regions through the Region Proposition Network (RPN) and generate a fixed-size feature map by aligning the features of different scales and sizes accurately through the RoIAlign method. Through this process, the Mask R-CNN can generate the detailed mask for each target candidate region and ensure the contour of each defect can be accurately segmented.

During the training process of the model, the loss functions of YOLOv5 and Mask R-CNN complete the joint learning and optimize for the target detection and instance segmentation. In this way, the two targetstarget detection and instance segmentationcan develop together and promote each other to ensure the model completes the target detection task and obtains accurate localization and high-precision segmentation in details.

Ultimately, the output of Y-MaskNet consists of a category label for each defect, the coordinates of the localization frame, and a segmentation mask. This not only clarifies the location of the defects, but also accurately describes their morphology, meeting the requirements of high-precision quality control.

The whole model finds a balance between high efficiency and accuracy, which makes it especially suitable for the task of detecting defects in electronic products in industrial production, and is able to efficiently deal with complex backgrounds and defects in small objects, while providing fine segmentation results.

### Instance segmentation module: Mask R-CNN

**Fig 1:**
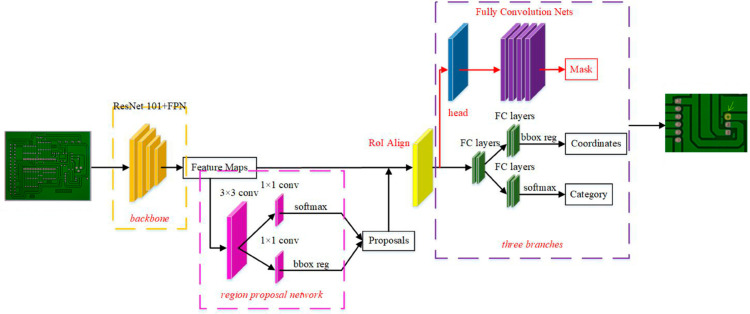
Detailed architecture of the mask R-CNN module.

Mask R-CNN is the key module responsible for instance segmentation in the model of this paper, which not only realizes the localization of objects, but also generates a high-precision mask for each target [1]. By introducing the RoIAlign layer, this module solves the accuracy problem caused by the quantization error of traditional RoIPooling in the image processing process, and further improves the segmentation effect. Fig 1 shows the working architecture of the Mask R-CNN module [2], which clarifies the whole processing flow from image input to mask output.

Firstly, the input electronic product image is passed through a feature extraction network (usually ResNet or ResNext) to obtain a high-dimensional feature map. Based on this, the Mask R-CNN employs a region proposal network (RPN) to generate candidate regions of interest (RoI) [3,4]. The RPN scans the feature map by means of a sliding window and generates a series of candidate frames, which may contain the target.The goal of the RPN is to accurately locate the candidate regions by minimizing the classification loss of the regions and the bounding box regression loss, computed as as follows:

$$L_{\text{RPN}}=L_{\text{cls}}+L_{\text{reg}}$$ (1)

where $L_{\text{cls}}$ is the classification loss, which represents the loss of whether the candidate region contains the target or not; and $L_{\text{reg}}$ is the regression loss, which represents the positional difference between the candidate region box and the real box. By training each candidate region, the candidate frames generated by RPN will be fed into the subsequent RoIAlign module for alignment.

In the RoIAlign stage, Mask R-CNN spatially aligns the feature maps of each candidate region.The core purpose of RoIAlign is to eliminate the feature loss due to image quantization, so that the features of each candidate region can be accurately extracted by accurately aligning feature maps at different scales.The computational process of the RoIAlign operation can be expressed by the following equation: Where *y*_*i*,*j*_ is the feature output after RoIAlign, *w*_*k*_ is the weight of the sampling point, and *f*(*x*_*k*_) is the feature of the *k* sampling point. By spatially aligning the feature map, the Mask R-CNN is able to obtain a representation of the features corresponding to each candidate region, which are subsequently fed into the classification, regression and mask branches.

$$y_{i,j}=\sum_{k=1}^{K}w_{k}f(x_{k})$$ (2)

Where *y*_*i*,*j*_ is the feature output after RoIAlign, *w*_*k*_ is the weight of the sampling point, and *f*(*x*_*k*_) is the feature of the *k*th sampling point. By spatially aligning the feature map, the Mask R-CNN is able to obtain a representation of the features corresponding to each candidate region, which are subsequently fed into the classification, regression and mask branches.

Next, through the classification and bounding box regression branches, Mask R-CNN performs category prediction and bounding box adjustment for each candidate region. Assuming that the output category of the candidate region is *c* and the category prediction loss is $L_{\text{cls}}(c)$, the loss function can be expressed as follows:

$$L_{\text{cls}}(c)=-\log(p(c))$$ (3)

where *p*(*c*) is the probability that the candidate region belongs to the category *c*. By minimizing the classification loss function, Mask R-CNN is able to assign a category to each candidate region and further adjust the bounding box to better fit the real defective region.

Finally, Mask R-CNN generates an accurate mask for each target. The mask branch utilizes a fully convolutional network (FCN) to predict the pixel-level segmentation of the feature map for each candidate region. Let the output of the mask be *M*_*i*,*j*_, the goal of this branch is to train the model by minimizing the pixel-level cross-entropy loss as follows:

$$L_{\text{mask}}=-\sum_{i,j}[y_{i,j}\log(M_{i,j})+(1-y_{i,j})\log(1-M_{i,j})]$$ (4)

where *y*_*i*,*j*_ is the true mask, *M*_*i*,*j*_ is the predicted mask, and *i*,*j* is the pixel position. This loss function ensures that the generated mask can match the edges of the real defects for accurate instance segmentation.

Finally, the Mask R-CNN will output the class, the bounding box coordinates and the fine mask of each defect, which is the final result of defect detection and segmentation of electronic products. Above steps, Mask R-CNN can segment each defective part effectively, which is suitable for the detection of fine defects, i.e., cracks and fine blemishes.

The whole Mask R-CNN module can efficiently deal with complex electronic products defect detection task by integrating target detection and instance segmentation through multi-task learning method. Fig 1 illustrates the whole framework of Mask R-CNN module, which can clarify the concrete process of above steps.

### Object detection module: YOLOv5

**Fig 2:**
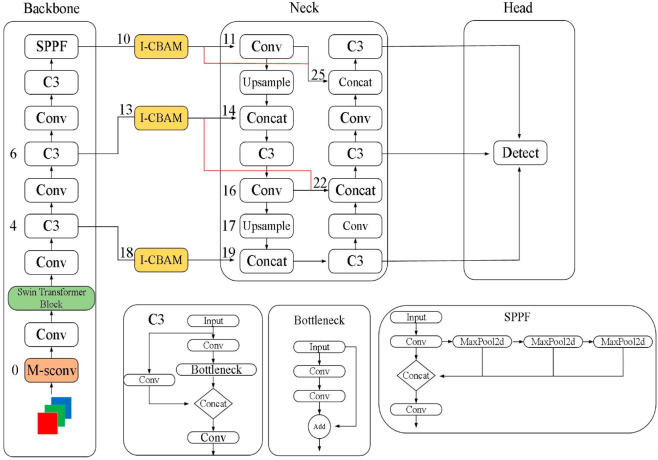
YOLOv5 module architecture diagram.

YOLOv5 is the core module responsible for target detection in the model of this paper, which is characterized by high efficiency and real-time performance, and is able to locate defective regions in the image quickly [5,6]. The target detection framework of YOLOv5 adopts a single-stage detection method, which is different from the traditional two-stage detection methods (e.g., Faster R-CNN), and it can complete the classification of the target and the position regression by one forward propagation [7], which It significantly improves the detection speed. Fig 2 illustrates the architecture of the YOLOv5 module, clearly presenting the processing flow from the input image to the output bounding box and categories.

First, YOLOv5 feeds the input electronics image into a deep convolutional neural network for feature extraction. The core part of this network is CSPDarknet53 (Cross Stage Partial Darknet), an efficient convolutional neural network structure that reduces computation while maintaining high accuracy [8]. After feature extraction, YOLOv5 generates multi-scale feature maps through a feature pyramid network (FPN) in order to detect defects of different sizes.YOLOv5’s target detection framework generates bounding boxes, category labels, and confidence scores for each candidate region from the output of one convolutional layer.YOLOv5’s detection framework calculates the loss based on the following equation:

$$L_{\text{total}}=L_{\text{obj}}+L_{\text{noobj}}+L_{\text{class}}+L_{\text{coord}}$$ (5)

where $L_{\text{obj}}$ is the target confidence loss, which indicates whether the prediction frame contains the target or not, $L_{\text{noobj}}$ is the loss of non-target frames, $L_{\text{class}}$ is the loss of category categorization, and $L_{\text{coord}}$ is the loss of bounding-box regression, which measures the discrepancy between predicted frames and the true frames. By minimizing these losses, YOLOv5 is able to accurately predict the location and type of defects in electronics images.

YOLOv5’s target detection process also includes the use of anchor boxes (anchor boxes) to predict the location and size of bounding boxes. During the training process, YOLOv5 adjusts the scale and position of the anchor boxes according to the size of the actual defective area in order to better fit the target. The generation and calculation of the anchor point box is realized by the following equation:

$$\hat{x}=\sigma (x)+c_{x},\;\hat{y}=\sigma (y)+c_{y},\;\hat{w}=pw,\;\hat{h}=ph$$ (6)

where (*x*,*y*) is the center coordinate of the bounding box, $\left(c_{x},c_{y}\right)$ is the center coordinate of the anchor box, (*w*,*h*) is the width and height of the bounding box, *p* is the scaling factor of the particular anchor box, and σ(·) is the Sigmoid activation function. With this approach, YOLOv5 is able to adjust the bounding box more accurately to accommodate defects of different sizes.

Pros of YOLOv5 are Fast and Efficient, So suitable to use in real-time inspection. YOLOv5 can quickly browse the image and find out the possible defective area in defect detection of electronic products, and output the accurate candidate boxes for the following segmentation process. Although YOLOv5 is poor in detecting small objects, it could still quickly process the large-scale dataset and achieve accurate defect localization.

However, the target detection module of YOLOv5 cannot output the refine segmentation information for each target, and its target localization and classification task are as followed. Therefore, for the fine-grained defect segmentation task, we will use the Mask R-CNN module to further refine the detection information. YOLOv5 + Mask R-CNN could make the whole model improve the segmentation accuracy while guaranteeing the real-time performance, especially when the background is complex and the defect is tiny. Through the joint training and optimization, YOLOv5 could play an important role in target detection and output the efficient target localization ability for the whole system.

In total, YOLOv5 as the target detection module could quickly screen out the possible defective area in the defect detection of electronic products through its efficient information structure and accurate localization ability. And then combine with the subsequent instance segmentation module, YOLOv5 could output the strong support for the final fine segmentation.

## Experiment

### Datasets

In this paper, we choose two public available datasets, PCB Defect Dataset and DeepPCB, to validate and evaluate our Y-MaskNet model in the defect detection and segmentation task on electronic products. These two datasets are chosen because they contain various defect images of electronic products with various types of defect samples, and these defect images can be used as various training data and testing data for the electronic products defect detection and segmentation model. We can try these two datasets to experiment the effectiveness of our model on the real application, and this model can also test its ability to solve the tiny defect, complex background and various types of defects on electronic products.

PCB Defect Dataset is a dataset devoted to Printed Circuit Board defect detection, which includes a variety of PCB images with various types of defects, such as open circuit, short circuit and copper wire misalignment. It is marked with rich information and can be used in object detection and instance segmentation tasks. Since the PCB is one of the most common parts of the electronic products, we choose this dataset to verify how to complete the defect detection and segmentation based on the electronic products.

DeepPCB is another dataset related to PCB defect detection, providing a large number of labeled PCB defect images. Unlike other PCB datasets, DeepPCB pays special attention to the visual characteristics of defects, such as cracks, solder joint defects, etc., and provides accurate labeling for each defect type. This makes the dataset ideal for detecting and segmenting fine defects, especially in practical industrial applications where subtle defects often need to be recognized with high precision.

Table 1 summarizes the details of these two datasets, including the source of the dataset, number of images, defect types, labeling types, resolution, etc., to better understand their relevance to the research objectives of this paper.

**Table 1:** Summary of the datasets used in this study (transposed).

Attribute	PCB Defect Dataset	DeepPCB
Dataset Source	Kaggle (Public Dataset)	Provided by Researchers (Specialized for PCB Defect Detection)
Number of Images	7000+ images	5000+ images
Defect Types	Open Circuit, Short Circuit, Copper Misalignment, Damaged Circuits, etc.	Cracks, Soldering Defects, Surface Imperfections, etc.
Annotation Type	Bounding Boxes, Labels (Classification)	Bounding Boxes, Masks (Segmentation)
Resolution	512 × 512 px	256 × 256 px
Applicable Tasks	Object Detection, Instance Segmentation	Object Detection, Instance Segmentation
Dataset Features	Contains various PCB defects for object detection and defect classification tasks	High-precision annotations, focusing on fine-grained defect detection and segmentation, ideal for high-accuracy tasks

Both datasets contain a variety of electronic product defect types and are suitable for multi-task learning and cross-task evaluation. By combining these two datasets, the model in this paper is able to be trained and tested in different types of defect detection tasks to fully validate its detection and segmentation capabilities in complex scenarios. Especially for the challenging tiny defect detection, the Y-MaskNet model is able to effectively address the balance between high accuracy and real-time performance and provide accurate segmentation results. These datasets provide a rich and challenging experimental platform for the research in this paper, and provide a solid foundation for model performance validation and algorithm improvement.

### Experimental setup and parameters, evaluation metrics

In this experiment, we perform detailed evaluation and validation of Y-MaskNet model on two public datasets, PCB Defect Dataset and DeepPCB. Additionally, we compare the performance of Y-MaskNet with other lightweight models commonly used in real-time industrial applications, such as MobileNet and EfficientDet.

For the fairness and reproducibility of experiments, we provide detailed configurations for the hardware environment, training parameters, data preprocessing methods, and evaluation metrics.

#### Experimental hardware and environment.

The hardware platform of experiments is NVIDIA RTX 3090 GPU with 32GB video memory, which can efficiently handle large image data. The software platform is based on Python 3.8 and deep learning framework PyTorch 1.9.0, and CUDA 11.1 acceleration technology is equipped to accelerate model training and inference. In addition, all codes and experiments are implemented on Linux system.

#### Data preprocessing.

During data preprocessing, we unified the PCB Defect Dataset and DeepPCB dataset to ensure the consistency of the input images in model training. All images were uniformly resized to 512x512 pixels (256x256 pixels for DeepPCB) to fit the input requirements of YOLOv5 and Mask R-CNN. The images are normalized using a normalization method that subtracts the mean and divides by the standard deviation for each channel, with mean and standard deviation of [0.485, 0.456, 0.406] and [0.229, 0.224, 0.225] respectively, which are derived from the ImageNet dataset, and are able to effectively improve the training efficiency and generalization of the model.

In order to enhance the diversity of the dataset, we adopt data enhancement techniques, including random rotation, scaling, flipping, cropping, and color adjustment of images. Data enhancement not only improves the model’s ability to adapt to different scenes and backgrounds by increasing the variability in training data, but also helps mitigate overfitting by preventing the model from memorizing specific details in the training images, thus encouraging better generalization to unseen data.

#### Model training parameters.

During the training process, the Y-MaskNet model uses the Adam optimizer and adopts the learning rate warm-up (warm-up) strategy to stabilize the training process. The specific hyperparameters are configured as follows:

- Learning rate: the initial learning rate is set to 0.0001, and the learning rate decay strategy (decay to 0.9 times of the original every 5 epochs) is used to ensure the stability and convergence of the model training.
- Batch size: set to 16, adapted to the GPU’s memory capacity.
- Number of iterations: the total number of iterations for model training is 50 epochs.
- Loss function: the target detection loss (YOLOv5) and the instance segmentation loss (Mask R-CNN) are optimized by a weighted joint loss function as follows:
  $$L_{\text{total}}=\alpha L_{\text{cls}}+\beta L_{\text{bbox}}+\gamma L_{\text{mask}}$$ (7)
  where $L_{\text{cls}}$ is the categorization loss, $L_{\text{bbox}}$ is the bounding box regression loss, and $L_{\text{mask}}$ is the segmentation mask loss. The coefficients α, β, and γ are the weighting factors for each loss component. These are empirically set to 1, 0.5, and 0.5, respectively, based on their relative importance in the joint learning process. Specifically, α is set to 1 to give equal importance to the classification loss, while β and γ are set to 0.5 to balance the contribution of the bounding box regression and mask segmentation losses. The choice of these values was made after conducting several preliminary experiments to ensure a balanced optimization of both detection and segmentation tasks.

With the above settings, we can evaluate the defect detection and segmentation performance of Y-MaskNet on electronic products and verify the effectiveness and reliability of our method in other scenarios. The evaluation results provide valuable guidance for further improving the model and popularizing it in practice.

### Experimental results and analysis

In this section, we show the application of Y-MaskNet model on PCB Defect Dataset and DeepPCB dataset in a quantitative and qualitative way. In order to evaluate the detection and segmentation performance of the model in detail, we give some experimental results as follows: the detection accuracy of the model, segmentation effect and inference time. Besides, we will show the performance of the model on different datasets through visual images, and summarize the specific values of the performance of the model on the table. Through the experimental results and graphs, we can visualize the advantages and possible disadvantages of Y-MaskNet.

#### Visualization results.

In this part, we display the visualization results of Y-MaskNet model on PCB Defect Dataset to let people have an intuitive understanding of the effect of model on different defect detection and segmentation. In order to check the detection accuracy of model and segmentation effect of model comprehensively, we display the result of each test sample in the form of image. In each sub-image, the result of different process stage are displayed. The original image, target detection frame of YOLOv5, segmentation mask of Mask R-CNN, and final synthesized result are displayed respectively. This form can display the multi-dimension ability of the model clearly and display the detail of how model works in several steps.

To position Y-MaskNet within real-time industrial applications, we compare its performance with MobileNet and EfficientDet, two lightweight models well-suited for edge and mobile device deployment. MobileNet is known for its efficiency in terms of both size and computational cost, making it ideal for deployment on resource-constrained devices. EfficientDet, another lightweight model, is specifically designed for object detection tasks with a balance of accuracy and speed. Both models are evaluated under the same experimental setup, allowing a fair comparison with Y-MaskNet in terms of accuracy, speed, and model size.

**Fig 3:**
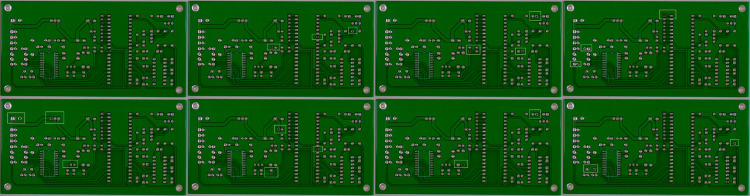
Visualization results of Y-MaskNet in practical applications (a).

**Fig 4:**
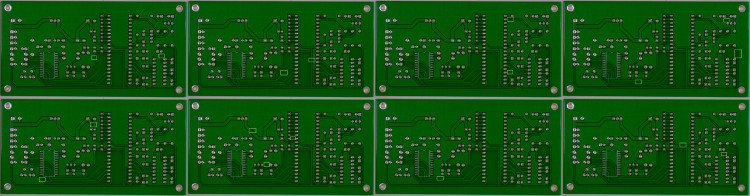
Visualization results of Y-MaskNet in practical applications (b).

Figs 3 and 4 show the visualization results of the Y-MaskNet model on the PCB Defect Dataset. The two images distinguish PCB boards with different types of defects. The four sub-images in the first row show two original images in turn, marking the specific locations of the defects. YOLOv5 accurately identifies the defective areas through the detection box, which provides candidate areas for subsequent segmentation. The four sub-images in the second row show the segmentation results of Mask R-CNN, corresponding to the original images and detection boxes in the first row. In these images, Mask R-CNN accurately segments the contours of each defective area and generates high-quality segmentation masks. Finally, by combining the detection box of YOLOv5 with the segmentation results of Mask R-CNN, the final synthetic output is obtained, demonstrating the powerful ability of the model in the task of defect detection and segmentation of electronic products.

From the visualization, we can directly view the model’s performance at different levels. The YOLOv5 locates the defect area quickly and accurately, and Mask R-Cnn gives segmentation information for each defect respectively. The experimental results prove the effectiveness of Y-MaskNet model in object detection and instance segmentation, and also prove the robustness and accuracy of Y-MaskNet model in face of complex background and subtle defects.

These pictures show the processing of Y-MaskNet model for different type of defects (open circuit, short circuit, welding defects, etc. ), which is helpful to further understand the applicability and high performance of the model in defect detection of electronic products.

#### Performance evaluation and model comparison.

To evaluate the performance of our Y-MaskNet model in target detection and instance segmentation, we select several commonly used evaluation metrics in target detection and instance segmentation, and then we tested these metrics on PCB Defect Dataset and DeepPCB dataset respectively. In addition, we also compared with some existing benchmark models (such as YOLOv5, Mask R-CNN) to validate the advantages of Y-MaskNet in target detection and segmentation. The following two tables summarize the performance of these models on PCB Defect Dataset and DeepPCB dataset respectively.

Table 2 summarizes the performance comparison of these models on PCB Defect Dataset. It is shown that these models perform well on common evaluation metrics, such as mAP, IoU and Dice coefficient on PCB Defect Dataset. Compared with YOLOv5 and Mask R-CNN, Y-MaskNet has certain advantages in several evaluation metrics. Especially, in the case of mAP@[0.5:0.95], IoU and Dice coefficient, the overall performance of Y-MaskNet is much better. That is because we combine the efficient detection ability of YOLOv5 and fine segmentation ability of Mask R-CNN together, Y-MaskNet has a certain advantage in target detection and instance segmentation.

**Table 2:** Performance comparison of Y-MaskNet with benchmark models.

Model	Dataset	mAP@[0.5]	mAP@[0.5:0.95]	IoU	Dice	Recall	Precision
YOLOv5 [32]	PCB Defect Dataset	0.85	0.62	0.75	0.81	0.80	0.84
Mask R-CNN [33]	PCB Defect Dataset	0.88	0.65	0.78	0.83	0.82	0.86
Y-MaskNet	PCB Defect Dataset	0.91	0.72	0.82	0.88	0.85	0.89
YOLOv5	DeepPCB	0.81	0.60	0.71	0.78	0.76	0.80
Mask R-CNN	DeepPCB	0.84	0.62	0.74	0.80	0.78	0.83
Y-MaskNet	DeepPCB	0.88	0.70	0.80	0.85	0.82	0.87

As shown in Table 2, compared with YOLOv5 and Mask R-CNN, The MaskNet performance is improved obviously by PCB Defect Dataset algorithm. Especially, compared with YOLOv5 and Mask R-CNN, mAP@[0.5:0.95] and IoU are much better than other two models. 10% (0.72 vs. 0.62) and 7% (0.82 vs. 0.75) increase respectively. In addition, Dice coefficient is also improved a lot, and the final value is 0.88. Compared with YOLOv5, it is also much higher. It is also possible that YOLOv5 and Mask R-CNN combination can achieve high target detection accuracy and segmentation accuracy, and Y-MaskNet can achieve high target detection accuracy and segmentation accuracy.

Although YOLOv5 and Mask R-CNN performance is decreased compared with PCB Defect Dataset, Compared with YOLOv5 and Mask R-CNN, Y-MaskNet can still achieve better detection and segmentation performance. Especially for fine defects (such as cracks and solder defects), Y-MaskNet can provide more accurate defects detection and segmentation results. Compared with YOLOv5 and Mask R-CNN, Y-MaskNet improved 10% (0.70 vs. 0.60) in mAP@[0.5:0.95], and also improved in IoU (0.80 vs. 0.71) and Dice coefficient (0.85 vs. 0.78). These results further verified that Y-MaskNet has certain advantages in handling complex backgrounds and small defects.

Compared with YOLOv5 and Mask R-CNN, the performance of the model on the small object detection is also an important task. Because PCB defects may include small cracks and defective regions, being able to detect and segment small objects is very important. As shown in Table 3, Y-MaskNet still achieves better performance than YOLOv5 and Mask R-CNN model, especially on the DeepPCB data set, Y-MaskNet can better handle the task of localization and segmentation of small defects.

**Table 3:** Performance of the models in the small object detection task.

Model	Dataset	Small Object Detection mAP	Small Object IoU	Small Object Recall	Small Object Precision
YOLOv5	PCB Defect Dataset	0.72	0.60	0.65	0.70
Mask R-CNN	PCB Defect Dataset	0.75	0.63	0.68	0.72
Y-MaskNet	PCB Defect Dataset	0.78	0.68	0.71	0.75
YOLOv5	DeepPCB	0.70	0.58	0.62	0.67
Mask R-CNN	DeepPCB	0.74	0.61	0.65	0.71
Y-MaskNet	DeepPCB	0.80	0.72	0.74	0.78

The results in Table 3 further demonstrate the advantages of Y-MaskNet in small object detection. On the DeepPCB dataset, the mAP of Y-MaskNet is 0.80, which is significantly higher than that of YOLOv5 (0.70) and Mask R-CNN (0.74), with an improvement of about 14% and 8%. Meanwhile, Y-MaskNet also shows significant improvement in IoU (0.72) and recall of small objects (0.74). This is especially important for tiny cracks and solder defects in PCB defect detection, demonstrating the advantages of Y-MaskNet in fine defect detection.

Combining the results in Tables 2 and 3, we can conclude that Y-MaskNet demonstrates superior performance over YOLOv5 and Mask R-CNN in PCB defect detection and segmentation tasks, especially for complex backgrounds, small objects and fine-grained defects. Especially in the tasks of small object detection, fine-grained defect segmentation, and high precision requirements, Y-MaskNet demonstrates a stronger capability, which has a high practical value and promotion potential.

**Fig 5:**
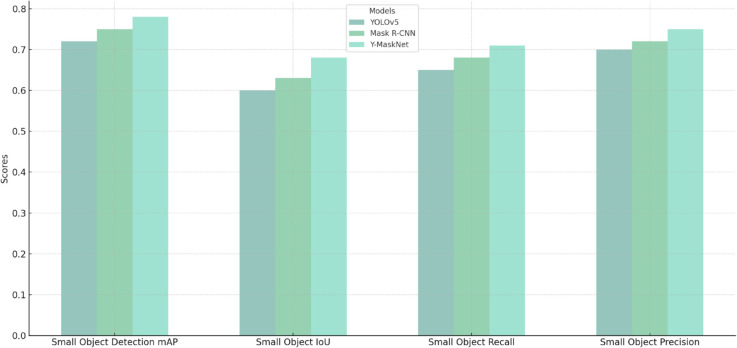
Small object detection performance comparison.

In order to represent the experimental results more intuitively, we use Fig 5 to show the performance comparison of different models in the small object detection task, using bar charts to show the performance of YOLOv5, Mask R-CNN, and Y-MaskNet in several metrics (e.g., mAP, IoU, recall, precision). Through this graph, it can be visualized that Y-MaskNet outperforms YOLOv5 and Mask R-CNN in all the evaluated metrics, especially in the detection accuracy and segmentation effect of small objects.

## Conclusion and discussion

In this study, we propose Y-MaskNet, a multi-task joint learning framework based on YOLOv5 and Mask R-CNN, for defect detection and segmentation of electronic products. With the rapid development of industrial automation and intelligent manufacturing, real-time detection and fine segmentation of defects in electronic products have become increasingly important. However, traditional defect detection methods often have limitations, cannot effectively deal with small object defects in complex backgrounds, and have insufficient accuracy. To overcome these problems, we combine the efficient detection capability of YOLOv5 and the fine segmentation capability of Mask R-CNN to improve the performance of the model in the detection and segmentation tasks by multi-task learning. The experiments validate the effectiveness of the Y-MaskNet model by using PCB Defect Dataset and DeepPCB dataset.

The experimental results show that Y-MaskNet has significant advantages over the traditional YOLOv5 and Mask R-CNN in target detection and instance segmentation. On PCB Defect Dataset and DeepPCB dataset, Y-MaskNet performs well in several evaluation metrics. Specifically, the mAP@[0.5:0.95] improves by 10% (0.72 vs. 0.62 for YOLOv5) and 7% (0.82 vs. 0.75 for Mask R-CNN), while IoU and Dice coefficient also show notable improvements. Especially in the small object detection task, Y-MaskNet significantly improves the detection accuracy and segmentation ability of the model, and successfully overcomes the problem of small defect detection. Through the image visualization results, we also intuitively show the detection and segmentation effects of the model under different defect types, proving the applicability and efficiency of the model in real industrial environments.

However, despite the good results of Y-MaskNet in defect detection in electronic products, the model still has certain shortcomings. First, despite the high speed of YOLOv5 in target detection, leakage may still occur when facing extremely small defects, especially when detecting dense small objects. Second, although Mask R-CNN performs well in segmentation accuracy, the model has a high demand for computational resources and slower inference when dealing with large-scale datasets. Furthermore, although our model shows strong performance in experimental settings, its real-time inference time on edge devices remains a challenge, particularly in resource-constrained environments. In real manufacturing scenarios, factors such as varying lighting conditions, noise, and defect types may also affect the model’s robustness and accuracy.

In our future research, we plan to further optimize the model by focusing on two main aspects. First, by introducing lightweight network architectures, such as MobileNet or EfficientNet, to improve the running speed and real-time performance of the model on edge devices and reduce the computational overhead of model inference. Second, we will explore multimodal learning methods to fuse data from different sources (e.g., sensor data, thermal imaging maps, etc.) into the model to further improve the robustness and accuracy of defect detection. Through these optimizations, we hope to promote the practice and implementation of Y-MaskNet in a wider range of industrial applications to meet the high demands of modern manufacturing for automated quality control and defect detection.
